# Wave kinetics of random fibre lasers

**DOI:** 10.1038/ncomms7214

**Published:** 2015-02-03

**Authors:** D V. Churkin, I V. Kolokolov, E V. Podivilov, I D. Vatnik, M A. Nikulin, S S. Vergeles, I S. Terekhov, V V. Lebedev, G. Falkovich, S A. Babin, S K. Turitsyn

**Affiliations:** 1 Aston Institute of Photonic Technologies, Aston University, Birmingham B4 7ET, UK; 2 Institute of Automation and Electrometry, Siberian Branch of the Russian Academy of Sciences, Novosibirsk 630090, Russia; 3 Novosibirsk State University, Novosibirsk 630090, Russia; 4 Landau Institute for Theoretical Physics, Russian Academy of Sciences, Chernogolovka 142432, Russia; 5 Moscow Institute of Physics and Technology, Dolgoprudny 141700, Russia; 6 The Budker Institute of Nuclear Physics, Novosibirsk 630090, Russia; 7 Weizmann Institute of Science, Rehovot 76100, Israel; 8 Institute for Information Transmission Problems, Moscow 127994, Russia

## Abstract

Traditional wave kinetics describes the slow evolution of systems with many degrees of freedom to equilibrium via numerous weak non-linear interactions and fails for very important class of dissipative (active) optical systems with cyclic gain and losses, such as lasers with non-linear intracavity dynamics. Here we introduce a conceptually new class of cyclic wave systems, characterized by non-uniform double-scale dynamics with strong periodic changes of the energy spectrum and slow evolution from cycle to cycle to a statistically steady state. Taking a practically important example—random fibre laser—we show that a model describing such a system is close to integrable non-linear Schrödinger equation and needs a new formalism of wave kinetics, developed here. We derive a non-linear kinetic theory of the laser spectrum, generalizing the seminal linear model of Schawlow and Townes. Experimental results agree with our theory. The work has implications for describing kinetics of cyclical systems beyond photonics.

## Abstract (web summary)

Kinetic theory is a mathematical framework that is used to describe non-linear systems with a large number of degrees of freedom. Here, the authors develop a concept of active wave kinetics of cyclic systems and describe the function of random fibre laser.

## Graphical abstract

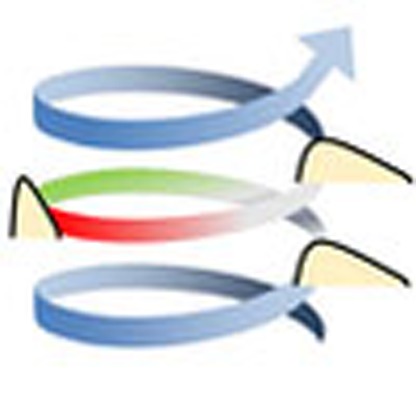

The mathematical description of non-linear systems with a large number of degrees of freedom far from thermodynamic equilibrium is still one of the major challenges of the modern theoretical physics that is also of great importance for various optical applications. The classical technique used in this field is kinetic equation approach that stems from the seminal work of Boltzmann^1^,^2^. Kinetic theory for waves has been pioneered by Peierls^3^, who derived the kinetic equation for phonons in anharmonic crystals applying averaging over random phases.

In many optical systems, key properties are defined by a large ensemble of light waves, randomized through optical noise, random scattering and non-linear interaction, which makes them a natural object for wave kinetic description. Moreover, an optical spectrum *F*(*ω*) is the correlation function directly described by the wave kinetic equation. Thus, the wave kinetic approach could provide a straightforward description for the slow relaxation of the optical spectrum to statistical equilibrium. In its classical form, the wave kinetics^4^,^5^ has recently been successfully applied to conservative non-linear optical systems^6^,^7^,^8^.

However, a number of practically important optical systems feature inherent gain and losses, often of a periodic nature. From the theoretical point of view, dissipation and amplification, that is, non-homogeneity over the evolution coordinate makes classical wave kinetic description of such dissipative (active) systems impossible.

The challenge becomes evident for a laser, one of the most interesting and arduous optical systems from a theoretical point of view. A vast number of various laser theories have been formulated for different gain mechanisms and cavity types (see, for example, refs 9, 10 and the references therein). However, most of them are dynamical and based on the representation of laser radiation via cavity modes. For cavity-free systems, such as random lasers^11^,^12^,^13^,^14^,^15^, dynamical models become complicated due to the co-existence of localized and extended modes and strong interactions between them^16^,^17^,^18^.

A fibre laser typically features a very large number of longitudinal modes weakly interacting via Kerr non-linearity. Such laser is, in essence, a natural active cyclic system having a double-scale kinetic evolution. Indeed, in fibre lasers due to gain and loss non-homogeneity over the spatial coordinate along the cavity, the fast evolution of the laser spectrum within each cavity round trip (cycle) is subject to strong changes. At the same time, the light evolves slowly from one round trip to another approaching some statistical equilibrium state. Owing to a complex nature of such kinetic processes, there is no yet a general theory that quantitatively describes spectra of radiation of fibre lasers, despite their wide practical use.

In this work, we introduce a new conceptual framework to describe cyclic wave kinetics of fibre lasers modelled by dissipative modifications of the integrable non-linear Schrödinger equation. This allows us to formulate the first-ever non-linear kinetic theory of laser radiation and describe, as an important practical example, an optical spectrum of a random fibre laser^19^. Since the system is close to integrable, we find very non-trivial kinetics, which makes for the conceptual novelty of our approach.

## Results

### Wave kinetic equation for fibre lasers

Kinetic theory describes an average (macroscopic) probabilistic evolution of complex system. Starting from basic microscopic dynamic equations governing the interaction of elementary constituents (for example, particles or waves), the kinetic description effectively reduces a large number of degrees of freedom in the original non-linear system by implying some assumptions about statistics of fluctuations. In the context of a wave system, the kinetic equation describes an evolution of quantities that are averages over times exceeding wave periods. In this case, waves with different frequencies acquire different phases enabling treating them as approximately independent at averaging. Nowadays, the wave kinetic approach (known in some contexts as wave turbulence^4^) is used in a range of physical applications varying from Bose–Einstein condensate to astrophysics^5^.

**Figure 1:**
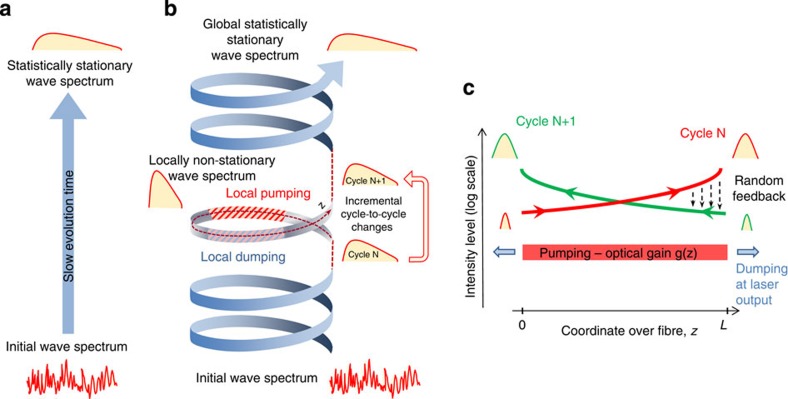
Wave kinetics in active cyclic systems. (**a**) In classical wave kinetics, initial wave spectrum evolves gradually to a statistically stationary wave spectrum when energy pumping/dumping is homogeneous over the evolution time. The evolution is governed by wave kinetic equation. The global statistically stationary wave spectrum is also a local stationary solution, that is, does not changed when shifted in time on any arbitrary value. (**b**) In active cyclic systems, the energy pumping/dumping act in a periodic way resulting in cycling dynamics and double-scale evolution of the wave spectrum. When the energy pumping/dumping changes within the cycle, the wave spectrum is locally non-stationary exhibiting strong changes within each cycle. This evolution is governed by a local pumping-driven wave kinetic equation equation (3). At the same time, the spectrum evolves in a gradual incremental way from cycle to cycle similar to classical wave kinetics. If the overall pumping within the cycle is equal to energy dumping, the system approaches the global stationary solution. (**c**) In a random fibre laser, the optical pumping is distributed over the fibre, while the dumping occurs at fibre ends where the radiation goes out. Each pass of the optical fibre is one cycle. The generation spectrum exhibits strong changes during evolution within each cycle because of optical gain. Random distributed feedback couples the optical spectrum on consequent cycles. As the gain is equal to losses in a laser, the optical spectrum must be identical on different cycles. Thus, the global stationary solution does exist.

In traditional wave kinetics, initial (arbitrary) wave spectrum *F*_*ω*_ evolves in a gradual way through cascades of numerous weak non-linear interactions towards a statistical steady state, as illustrated by Fig. 1a. The evolution from initial to asymptotically stationary spectrum is governed by the wave kinetic equation^4^. The long-time asymptotic state is steady both globally and locally meaning that statistical properties do not change at any arbitrary time shift. This is achieved when the external pumping of energy into the system and dumping of energy out of the system are time independent.

In fibre laser, radiation undergoes strong periodic (cyclic) changes along the cavity due to combined action of gain, loss, dispersion and non-linearity, superimposed on a slow evolution from one round trip to another. The global statistical equilibrium still may exist in terms of the Poincare mapping^20^ as a state that is statistically reproduced after each round trip. However, contrary to the case of systems homogenous over evolution variable, a local statistically steady state does not exist, meaning that the wave spectrum is changed substantially at any arbitrary time shift. As a result, two scales over evolution coordinate do exist in laser systems—a fast evolution within each round trip, and slow evolution from cycle to cycle. This results in non-uniform spiral-like evolution that differs from a monotonic relaxation in classical wave kinetics, as schematically depicted in Fig. 1b. It is important that the amplitudes of waves could be sufficiently increased or decreased during the fast evolution within each round trip depending on either the pumping or dumping being dominant at particular moment in a laser cavity. So the classical wave kinetic approach cannot be directly applied to the typical fibre lasers as the strong local (quasi periodic) dynamics of the wave spectrum must be taken into account.

To deal with active cyclic systems, we derive a pumping-driven wave kinetic equation governing the fast and strong wave spectrum evolution within round trip, in addition to the ‘standard’ wave kinetic equation governing the slow and incremental wave spectrum evolution from cycle to cycle.

Further, we briefly outline the derivation of the kinetic equation for the cyclic active wave system having numerous random-phase waves non-linearly interacting via cubic non-linearity, which is assumed to be weak. We consider the quadratic dispersion law specific for optics and spectrally narrow excitations. The system is under periodic energy pumping and dumping repeated in a cyclic way and is evolving within each period and from cycle to cycle Fig. 1b.

We start from the generic dynamical equation describing the evolution of the complex envelope field *ψ* over the evolution coordinate *z* (that, for instance, can be a propagation distance inside the fibre cavity) in dispersive non-linear medium^21^:


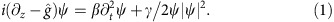


Here *t* stands for time, while *z* is the evolution coordinate within the cycle. For the wave system, coefficients *β* and *γ* describe, respectively, the dispersion and non-linearity of the running wave with intensity proportional to 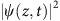, the linear operator 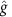 describes energy pumping/dumping into/from the system. In the particular case of optics, equation (1) is the generalized non-linear Schrödinger equation, which describes light propagation in one-dimensional media, *γ* is associated with Kerr non-linearity and 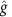 is the operator describing an optical spectrally dependent gain. The dynamical models based on the non-linear Schrödinger equation (1) are widely used for describing laser radiation in fibre lasers (see refs 22, 23), as well as other optical systems characterized by strong influence of noise and stochasticity^24^,^25^,^26^.

The conservative part of equation (1), that is, one-dimensional NLSE, is completely integrable and possesses an infinite number of the integrals of motion^27^. In the context of kinetic consideration, this equation presents a very special case. As was pointed out by Zakharov^27^, there is a wide class of integrable equations leading to trivial kinetics, that is, no kinetics at all, and the one-dimensional NLSE belongs to the class. The deviations from the integrability in equation (1) are given by the term with 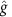 and are of crucial importance since; indeed, these deviations determine a non-trivial kinetics of the wave system. We construct a general scheme enabling the derivation of kinetic equation for such nearly integrable systems (see Supplementary Note 1 and Supplementary Fig. 1) that can be employed in a variety of other physical applications.

The wave kinetic theory deals with a pair correlation function


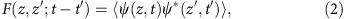


here angle brackets denote averaging over *z* larger than the dispersion length 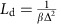, where Δ is the characteristic width of the wave spectrum defined as a Fourier transform of the one-point correlation function *F*(*z*, *z*, *t*): *F*_*ω*_(*z*)=∫*dt e*^*iωt*^ *F*(*z*, *t*). Further we are interested in kinetics, that is, in the evolution of a wave spectrum. Note that in optics, *F*_*ω*_(*z*) corresponds to the directly measured optical spectrum.

Next we derive the wave kinetic equation on correlation function *F*_*ω*_ using the deviation of equation (1) from the integrable NLSE. We use a standard assumption that the field *ψ* consists of numerous waves with random phases. These waves interact via small non-linearity that is the origin of their randomness. Extensive technical details of how the local wave kinetic equation is derived using the diagram techniques can be found in Supplementary Notes 2 and 3.

The resulting wave kinetic equation reads:


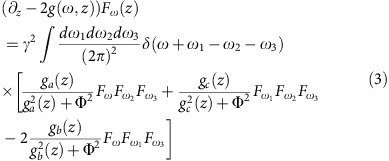


Here *g*(*ω*,*z*) is a frequency dependent gain, 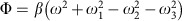
is a dispersion factor, *g*_*a*_=*g*(*ω*, *z*)+*g*(*ω*_2_, *z*)+*g*(*ω*_3_, *z*)−*g*(*ω*_1_, *z*), *g*_*b*_=*g*(*ω*, *z*)+*g*(*ω*_1_, *z*)+*g*(*ω*_3_, *z*)−*g*(*ω*_2_, *z*), *g*_*c*_=*g*(*ω*_1_, *z*)+*g*(*ω*_2_, *z*)+*g*(*ω*_3_, *z*)−*g*(*ω*, *z*). The equation is derived under double separation of scales, that is, we consider the case of strong dispersion over spectrum compared with gain, and we assume that the gain itself if much larger than gain variation over the spectrum width. Note that equation (3) describes wave kinetics in a rather general class of active cyclic system not limited to quadratic dispersion relation: different dispersion laws can be easily incorporated in (3) by a corresponding change of the factor Φ.

There are several principal differences between the wave kinetic equation (3) for the active cyclic system and a classical wave kinetic equation. First of all, as the energy pumping and dumping is non-homogenous over the evolution coordinate *z* within the cycle, the wave spectrum exhibits strong changes during the evolution within the cycle, so the local stationary statistically steady state does not exist. Formally that means one cannot equate the derivative over evolution coordinate *z* to zero and find in this way a stationary solution similar to classical wave kinetics.

The traditional wave kinetic equation for a conservative Hamiltonian system could be obtained from the local wave kinetic equation (3) in the limit of zero gain. In this case, the gain-related Lorentzian terms 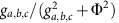 in the right-hand side of equation (3) turn into delta-functions, ensuring the energy conservation and giving the classical kinetic wave equation. However, the classical kinetic equation derived from NLSE is trivial in a sense that the right-hand side vanishes because of integrability of one-dimensional NLSE^27^. In other words, in NLSE-based integrable system, the spectrum does not evolve at all. This is not the case for local wave kinetic equation (3). From the formal point of view, wave interaction is uniform over slow evolution time^4^ in classical wave kinetics. In active cyclic systems, however, these interactions are mediated by a non-homogenous gain (see Lorentzian factors due to gain in equation (3)), which results in the interaction being effective over the finite interval of the evolution coordinate only. Note that equation (3) does not conserve momentum, because the pumping and dumping are changed over the evolution coordinate *z*. We would like to also note that an interesting modification of the conventional wave turbulence kinetic equation was studied in the paper by Ascheri *et al*.^28^, where the integrability of the original NLSE model was broken by the transverse spatial inhomogeneity of the refraction index.

### Non-linear kinetic theory of a laser’s optical spectrum

Now apply the formalism of wave kinetics in active cyclic systems to an optical system and derive for the first time, to the best of our knowledge, a non-linear kinetic theory of the optical spectrum of a laser. In general, the classical wave kinetics cannot be applied to describe the laser optical spectrum, because lasers are active cyclic systems with double-scale evolution. The first steps towards kinetic consideration have been made in the experimental works^29^,^30^, in which the challenge was outlined. First, a heuristic analysis was performed for a specific system, and strong experimental evidence of the need for new rigorous non-linear kinetic theory was presented there^29^,^30^.

Here the local wave kinetic equation (3) allows us to formulate a non-linear kinetic theory of the laser spectrum generalizing the famous linear kinetic theory by Schawlow and Townes^31^. We focus on a particular, albeit very interesting case of the random fibre laser operating via Rayleigh scattering feedback^19^. Until now, apart from a straightforward NLSE-based numerical modelling^32^, there was no theory, neither dynamical nor kinetic, describing the optical spectrum of the random fibre laser, which was an additional motivation to choose this particular system as a test-bed of our general theory.

Consider a random fibre laser of length *L* that is an optically pumped long span of a standard telecommunication fibre. Weak random distributed feedback is caused by the Rayleigh scattering in a fibre core on refractive index inhomogeneities, and the Raman gain is induced by the pump radiation, jointly resulting in random lasing^19^. The laser’s optical spectrum consists of numerous waves of random amplitudes and phases which interact via Kerr non-linearity. Effectively, the cavity of the random fibre laser corresponds directly to the concept of the active cyclic systems, Fig. 1c. Indeed, there are two counter-propagating generating waves that are identical under the condition of symmetric optical pumping. The propagation of each wave from one end of the optical fibre to another end is governed by the dynamical equation (1). We treat each pass of the optical fibre as one kinetic cycle. During the propagation, the optical spectrum is subject to strong changes. Indeed, there is a distributed optical gain all over the fibre, and the generation intensity at the initial point of each cycle, at *z*=0, is very low since almost all optical power is emitted from the fibre end. Therefore, the local wave kinetic equation (3) must be applied.

**Figure 2:**
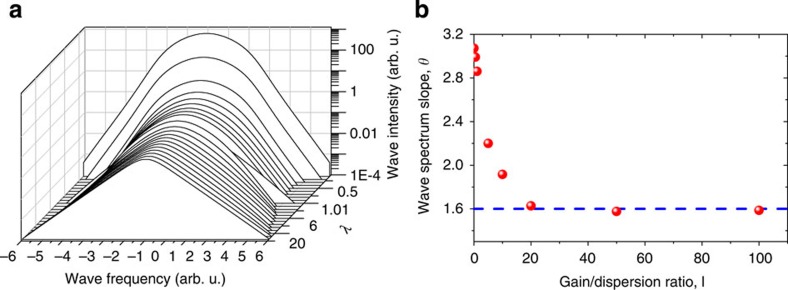
Statistically steady-state solutions of the local wave kinetic equation. The spectra are numerically calculated from the local wave kinetic equation (3) in a particular case of a random fibre laser as an example Supplementary Equation 43. (**a**) Wave spectrum depending on gain/dispersion ratio *λ*=2*g*/*β*Δ^2^, where Δ is the optical spectrum width. (**b**) Wave spectrum slope *θ* at different gain/dispersion parameter *λ*. The slope is defined from the approximation of the wave spectrum wing by exponential function, exp(*−θx*). Dotted line shows the wave spectrum slope in the case of hyperbolic secant shape wave spectrum.

We focus on a statistical equilibrium corresponding to an average laser spectrum that is approached over many round trips. Formally, the existence of the steady-state laser spectrum is equivalent to the following boundary condition on correlation function: 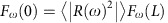, where 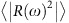 is the random reflection coefficient of the laser output coupler. Taking the local kinetic equation (3) and the boundary conditions, using the perturbation theory for a small non-linearity and after some calculations, we derive the kinetic equation directly governing steady-state laser spectrum *F*_*ω*_, (Supplementary Note 4 and Supplementary Equation 43), being just a modified version of the local kinetic equation (3). Depending on the value of the dispersion, the wave spectrum, that is, the generation spectrum of the random fibre laser, has a different shape Fig. 2a, and different rate of energy transfer from spectrum centre to spectrum wings Fig. 2b. Note that in the case of long Raman fibre laser with a conventional linear cavity, similar hyperbolic secant spectrum shape was observed and introduced in a phenomenological way in the limiting case of the dispersion being much larger than non-linearity^29^.

**Figure 3:**
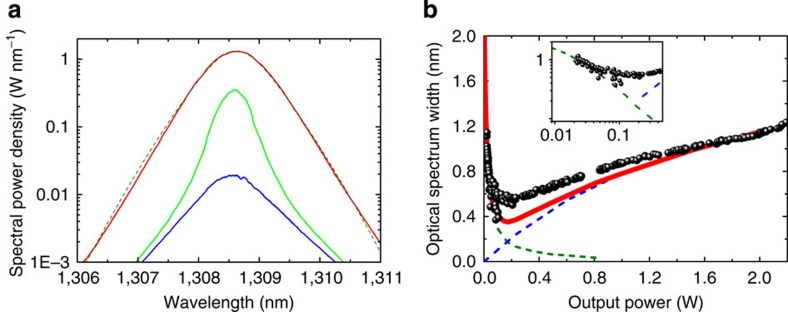
Non-linear kinetic description of the random fibre laser optical spectrum. (**a**) Experimentally measured optical spectrum: near the generation threshold (blue curve, laser power=0.025 W), slightly above the generation threshold (green curve, 0.2 W) and well above the generation threshold (red curve, 1.5 W). The optical spectrum predicted by the local wave kinetic equation Supplementary Equation 43, for laser power 1.5 W is shown by dashed red line (**b**) Spectrum width as a function of the laser’s output power in theory and experiment. Experimental data are shown by black circles. The prediction for spectrum broadening from non-linear kinetic theory based on local wave kinetic equation (3) is shown by blue dashed line. The prediction for spectrum narrowing from modified linear kinetic Schawlow–Townes theory Supplementary Equation 47, is shown by dashed green line. Red line is a sum of non-linear and linear contributions. Inset—spectral narrowing near the threshold in a logarithmic scale.

To verify the predictions of our non-linear kinetic theory on the laser’s spectrum, we designed the random fibre laser operating in the same domain of parameters as in the theory (see Methods and Supplementary Note 5 and Supplementary Fig. 2). The laser spectrum was measured and is shown in Fig. 3a. Below the threshold, we observe an amplified spontaneous emission spectrum that corresponds to the gain profile. High above the generation threshold, the random fibre laser spectrum demonstrates non-linear kinetic broadening. The spectrum shape is perfectly described by our theory Fig. 3a. Moreover, the theory describes the laser’s spectrum broadening law over the pump power Fig. 3b (red line).

Besides the non-linear kinetic contribution to the wave spectrum, well pronounced at high power, there is also a linear kinetic contribution dominant at low powers. Indeed, it is well known from the seminal work of Schawlow and Townes^31^ that the laser spectrum exhibits spectral narrowing while the pump power increases above the generation threshold. The spectral narrowing is observed in random fibre laser spectrum as well (see Fig. 3b. However, the approach of Schawlow and Townes^31^ can be used only for laser cavities having distinct well-defined cavity modes, which is not the case for the random fibre laser having modeless, that is, continuous spectrum. To deal with that, we modify the approach of Schawlow–Townes and derive the equation describing spectrum narrowing of the random fibre laser at low power (see Supplementary Fig. 3, Supplementary Note 6 and Supplementary Equation 45). Its solution, Supplementary Equation 47, describes well the experimentally observed optical spectrum narrowing near the generation threshold Fig. 3b, inset.

Note that as a matter of fact, the Supplementary Equation 45 is a linear kinetic equation valid at lower powers where one can neglect the non-linear interactions between different spectral components. In this sense, the local wave kinetic equation, equation (3), describing the non-linear spectrum broadening high above the generation threshold, is the extension of the Schawlow–Townes equation to the non-linear mode interaction case. The real optical spectrum has both—linear and non-linear—contributions. The sum of linear and non-linear terms describes well the experimentally measured laser spectrum width in all power range Fig. 3b. The small residual difference between the theory and the experimental data could be attributed to the influence of the pump wave induced cross-phase modulation (XPM) effect. The estimate gives the value of XPM induced spectral width of 0.2 nm at low power.

## Discussion

The general formalism of wave kinetics of active cyclic systems presented here could be applied for various optical systems where stochasticity is important, such as random lasers of other types, lasers with open or unstable resonators, multi-mode lasers with very large number of generation modes and other systems. However, our theoretical formalism for statistics of cyclic active systems can have implications far beyond photonics. To give the example most close to heart: within every heartbeat from diastole to systole, there are substantial and complicated changes of the blood flow—when one starts to run, it takes many heartbeats for a system to evolve into a new state. The world is full of such systems, non-Hamiltonian in their nature, which evolve to the statistical equilibrium in cycles as external energy pumping and dumping occurs in a periodic way; for instance, day and year cycles in meteorology, long-haul fibre transmission links, lasers, Rayleigh–Taylor instabilities in various media (water, atmosphere, coatings in surfaces). Our work shows a direction for describing such systems—considering slow evolution of the respective correlation function averaged over several cycles.

## Methods

### Experimental set-up

In brief, the random fibre laser comprises a pump source at 1,115 nm coupled to 850 m of a phosphosilicate fibre. The laser generates near 1,308 nm. We minimize the length of the random fibre laser to keep the pump nearly undepleted and maintain a low non-linearity factor, which is actually the ratio of the generation power to the pump power. The shorter the length, the higher the generation threshold, and, therefore, the higher pump power at given generation power. To compare the non-linear kinetic laser theory predictions with experimental data, we measure all fibre parameters except non-linearity coefficient that is known from the literature. The coefficients are linear losses *α*=0.09 km^–1^, dispersion coefficient *β*=4.3 ps^2^ km^–1^, *γ*=7 (km W)^–1^, Raman gain coefficient *g*_R_=0.68 (kmW)^–1^.

## Additional information

**How to cite this article:** Churkin, D.V. *et al*. Wave kinetics of random fibre lasers. *Nat. Commun.* 6:6214 doi: 10.1038/ncomms7214 (2015).

## Supplementary Material

Supplementary Information Supplementary Figures 1-3, Supplementary Notes 1-6 and Supplementary References
