# Publisher Correction: Wave kinetics of random fibre lasers

**DOI:** 10.1038/ncomms16190

**Published:** 2018-05-25

**Authors:** D. V. Churkin, I. V. Kolokolov, E. V. Podivilov, I. D. Vatnik, M. A. Nikulin, S. S. Vergeles, I. S. Terekhov, V. V. Lebedev, G. Falkovich, S. A. Babin, S. K. Turitsyn

Nature Communications 6: Article number: 6214; DOI: 10.1038/ncomms7214 (2015); Published 02 03 2015; Updated 05 25 2018

The original HTML version of this Article had an incorrect volume number of 2; it should have been 6. This has now been corrected in the HTML; the PDF version of the Article was correct from the time of publication.

