# Supplementary material for: Wave kinetics of random fibre lasers
Source: Nat Commun. 2015 Feb 3;6:6214. doi: 10.1038/ncomms7214 (PMC4347200; doi:10.1038/ncomms7214)
Supplement: Supplementary Information — Supplementary Figures 1-3, Supplementary Notes 1-6 and Supplementary References [file ncomms7214-s1.pdf]

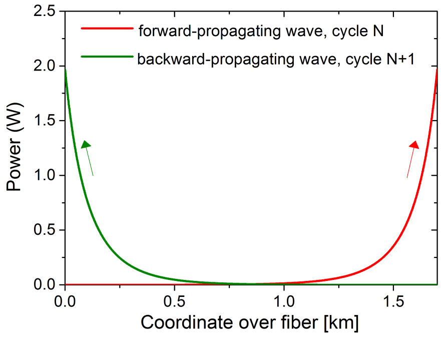

Supplementary Figure 1: Longitudinal power distribution over the random fibre laser.

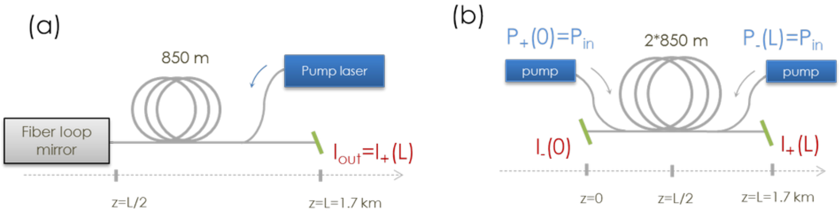

Supplementary Figure 2: Random fibre laser. (a) The experimental setup. (b) The equivalent configuration of twice the length.

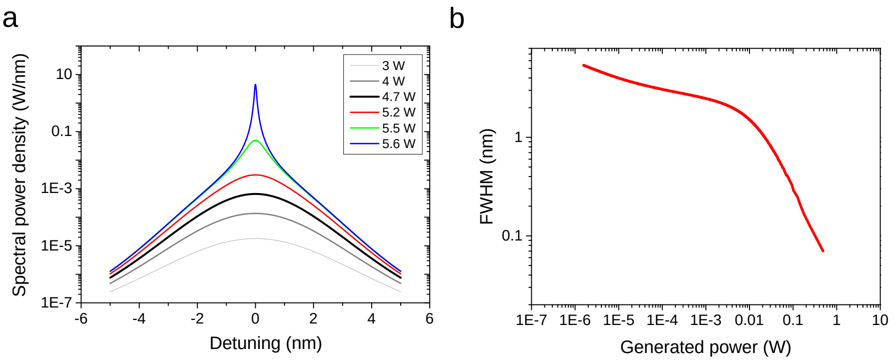

Supplementary Figure 3: (a) Wave spectrum at different power levels (b) Spectral full width at half maximum. The data are numerically calculated from Eq. (47).

Supplementary Note 1. Basic dynamic equations

Here, we provide details of the formalism for describing wave kinetics in active cycled systems. We illustrate our method with an example of a random distributed feedback fibre laser. However, the approach is general. The random laser here is a span of optical fibre of length L that is optically pumped from fibre ends. The randomly backscattered light in the waveguide is amplified by the Raman effect, and the system starts to lase. See more about random fibre lasers in the recent review [1].

A schematic distribution of the generated wave power along the fibre span is presented in Supplementary Figure 1. Two electromagnetic waves, – propagating to the right and the left, are generated in the fibre. Due to amplification, their amplitudes are increased during the propagation and achieve maxima near the ends of the fibre, before passing outside the fibre. Let us stress that nonlinear interaction between counter-propagating waves is weak, since their amplitude maxima are achieved at opposite ends of the fibre. Therefore, they can be considered independently.

We begin with the dynamic model describing the evolution of the envelope of the generation electromagnetic field, ψ, over the coordinate z within the fibre, at $0 < z < L$, in which L is the span length. The equation for the generation wave propagating in the fibre to the right is

$$i\left(\partial_z - \hat{g}\right)\psi = \beta\partial_t^2\psi + \frac{\gamma}{2}\psi|\psi|^2, \tag{1}$$

in which t stands for time, γ is the Kerr nonlinear coefficient, and β is the quadratic dispersion coefficient. We consider the generation processes high above the threshold, so we neglect noise terms in Eq. (1). An equation similar to Eq. (1) describes the signal propagating to the left. The only difference is in the sign of the derivative ∂_z.

The effective gain operator $\hat{g}$ is determined by the energy pumping and attenuation of the signal. In the frequency domain, it is a frequency-dependent function $g = g_R P(z) - \alpha$, in which g_R is the Raman gain coefficient, $P(z)$ is the power of the pumping wave, and α is the linear attenuation coefficient in the fibre. The distribution of energy pumping over the evolution coordinate z is defined by the factor $P(z)$ that is assumed to be known (it can be consistently found from the power balance equations). Lasing occurs for frequencies near the maximum of the effective gain g. We can approximate spectral dependence of the effective gain by the Taylor expansion of the gain coefficient near its maximum:

$$g(\omega) = g_R P(z) - \alpha - aP(z)\omega^2 \tag{2}$$

Further we use a notation $g_0 = g_R P(z) - \alpha$. Note that above the generation threshold,

the condition $g_0 > 0$ must be fulfilled.

In reality, the power of the pumping wave $P(z)$ depends on the generation wave ψ. They are related via the power balance equations [2, 1]. Therefore, the problem should be solved in two steps. First, we must find ψ at a given P and then self-consistently find $P(z)$, using the balance equation. Here, we concentrate on the most challenging first step.

In a random fibre almost all generated radiation goes out from the fibre end. Only a small part of the energy is reflected back via weak Rayleigh back scattering processes. Due to spatial distribution of the gain, amplitudes of generated waves are increased during evolution, making the scattering process feedback most effective only at the very end of the fibre. This implies an effective initial condition for the generation wave ψ_+, propagating to the right, through the amplitude of the generation wave ψ_-, propagating to the left. Formally, the effective initial conditions for the waves can be introduced as

$$\psi_+(0,t) = \hat{R}_l \psi_-(0,t), \quad \psi_-(L,t) = \hat{R}_r(\omega)\psi_+(L,t), \tag{3}$$

in which R_l and R_r – reflection coefficients on the respective left end and the right end of the fibre are defined in the frequency domain. These coefficients may have different ω-dependence in different schemes. In the case of the random fibre laser, $|R| \ll 1$, the reflection smallness leads to the conclusion that the signal is only weakly disturbed by the reflection, thus justifying the conditions (3).

The spectrum of the generated wave in the random fibre laser is relatively broad (in comparison to few-mode lasers) and consists of a high number of spectral components near the carrying frequency ω_0 (see [1]). The main challenge here is to describe an influence of non-linearity on the generation spectrum. To do that, we use the standard kinetic approach dealing with averaged quantities. We assume that the dispersion length $(\beta\Delta^2)^{-1}$ (in which Δ is the spectral width) is small in comparison to the fibre length L. Then phases of the harmonics with different frequencies possess essentially different phases. Therefore, at averaging over a length larger than the dispersion length the harmonics can be treated as approximately independent.

The main object in kinetic theory is the pair correlation function

$$\langle \psi(z, t_1 + t)\psi^\star(z, t_1)\rangle = \int \frac{d\omega}{2\pi} \exp(-i\omega t) F(z, \omega), \tag{4}$$

in which angular brackets mean averaging over distances larger than the dispersion length and $\psi^\star$ designates complex conjugation. Due to assumed time homogeneity, the average (4) solely depends on the time difference t and is independent of t_1. However, when

examining real fibres, it is useful to average over time (integrate over t_1) to eliminate effects related to different fluctuations (noises) neglected in our formalism. Let us stress that due to z-dependence of the generation wave, the system is not homogeneous in space, in contrast to the time behavior. The function F is nothing else but the spectrum of the generated signal. Note that the signal intensity I can be expressed via the spectrum, it is the integral

$$I \equiv \langle |\psi|^2 \rangle = \int \frac{d\omega}{2\pi} F(\omega). \tag{5}$$

The boundary conditions (3) lead to the following relations for the averages

$$F_+(0,\omega) = |R_l(\omega)|^2 F_-(0,\omega), \quad F_-(L,\omega) = |R_r(\omega)|^2 F_+(L,\omega), \tag{6}$$

in which F_+ and F_- correspond to the generation waves respectively propagating to the right and the left. Further, we consider the symmetric stationary situation in which $R_l = R_r$ and $F_+(z) = F_-(L-z)$. Then we come to the condition

$$F(0,\omega) = |R(\omega)|^2 F(L,\omega), \tag{7}$$

for the signal propagating to the right. The condition relates values of the correlation function F taken at different ends of the fibre. Therefore, it can be considered as a closed loop.

Supplementary Note 2. Kinetics

To examine non-linear effects in the random laser, a perturbation theory must be developed. The starting point for the theory is the basic equation (1) for the envelope $\psi(z,t)$. We treat the non-linear term in Eq. (1) as a perturbation and expand the solution of the equation over the non-linearity. Then we use the expansion for calculating the average (4). Effectively, we apply a diagrammatic technique of the type developed first by Wyld [3] in the context of hydrodynamic turbulence. The diagrammatic technique enables us to calculate correlation functions of the field ψ.

Below, we aim to derive an evolution equation for the spectrum F. The equation enables us to analyze the form of the spectrum and its dependence on the system parameters. Our derivation is performed in the spirit of the derivation of the standard kinetic equation [4, 5] for classic waves. However, our system is close to an integrable one since at $g = 0$, the basic equation (1) is the non-linear Schrödinger equation that is completely integrable and possesses an infinite number of integrals of motion. There are no kinetics in the system of waves described by the non-linear Schrödinger equation [6]. Therefore,

non-trivial kinetics is related to the presence of the gain term g that makes the situation qualitatively different from the standard kinetic equation and requires a consistent derivation of a generalized kinetic equation.

A formal solution of the equation (1) can be written as

$$\psi(z,t) = \int dt'\, G(z,z_\star,t-t')\psi(z_\star,t')$$
$$-\frac{i\gamma}{2}\int dt' \int_{z_\star}^{z} dz'\, G(z,z',t-t')\psi(z',t')|\psi(z',t')|^2, \tag{8}$$

in which $z_\star$ is an arbitrary point and

$$G_0(z,z',t) = \theta(z-z')\int \frac{d\omega}{2\pi}\exp\left[-i\omega t + \int_{z'}^{z} dz''\,(g+i\beta\omega^2)\right] \tag{9}$$

is the Green function determining a linear response of the system to an external influence. Analogously, it is possible to consider the "back" in z evolution. For example, in the linear approximation

$$\psi(z',t') \approx \int dt\; G(z,z',t-t')\psi(z,t). \tag{10}$$

As a next step, we derive an equation for the function F (4). Eq. (1) yields

$$\partial_z F(z,\omega) = 2gF - \frac{i\gamma}{2}\int dt\;\exp(i\omega t)$$
$$\left\langle \psi(z,t)\psi^\star(z,0)\left[|\psi(z,t)|^2 - |\psi(z,0)|^2\right]\right\rangle. \tag{11}$$

Here, as above, the angular brackets mean averaging over distance larger than the dispersion length. The equation (11) implies that both g and F are slowly varying at the length of averaging. We assume $\beta\Delta^2 \gg g_0$. We can then choose the averaging length l much smaller than g_0^{-1}. In addition, the inequality $aP\Delta^2 \ll g_0$ must be satisfied as a manifestation of the spectrum narrowness in comparison with the characteristic frequency range of the Raman scattering. Therefore, we come to the chain of the inequalities $aP\Delta^2 \ll g_0 \ll \beta\Delta^2$ that must be satisfied for our theoretical scheme to be valid.

The phase randomization caused by dispersion leads to approximately Gaussian statistics of the field ψ, since it appears to be a sum of a large number of independent terms. Therefore, by calculating averages like (11), we can use the Wick theorem (the presentation of the average of some product of ψ fields via its pair correlation functions). However, application of the Wick theorem to the combination on the right-hand side of Eq. (11) gives zero. Therefore, we must take into account a weak correlation between different harmonics caused by non-linearity. Technically, one should exploit the non-linear contri-

bution to ψ from Eq. (8)

$$\psi_{non}(z,t) = -\frac{i\gamma}{2}\int dt' \int_0^z dz'\, G(z,z',t-t')$$
$$\int dt_1\; G(z,z',t_1-t')\psi(z,t_1)\, \left|\int dt_2\; G(z,z',t_2-t')\psi(z,t_2)\right|^2 , \qquad (12)$$

in which we substitute the expression (10). The goal of the substitution is to express $\delta\psi$ in terms of $\psi(z,t)$. Then averages in the right-hand side of Eq. (12) are expressed in terms of the function $F(z,\omega)$ (4).

Using the Wick theorem, substituting the expression (9) and taking integrals over time, we obtain from Eq. (12)

$$(\partial_z - 2g)\, F = \gamma^2 \int \frac{d\omega_1 d\omega_2 d\omega_3}{(2\pi)^2}\delta(\omega+\omega_1-\omega_2-\omega_3)$$
$$\left[\frac{g_a\, F F_2 F_3}{g_a^2+\Omega^2} + \frac{g_c\, F_1 F_2 F_3}{g_c^2+\Omega^2} - \frac{2g_b\, F F_1 F_3}{g_b^2+\Omega^2}\right] . \qquad (13)$$

Here, $F = F(z,\omega)$, $F_1 = F(z,\omega_1)$, so the following notations are introduced

$$\Omega = \beta(\omega^2+\omega_1^2-\omega_2^2-\omega_3^2), \quad g_a = g(\omega)+g(\omega_2)+g(\omega_3)-g(\omega_1),$$
$$g_b = g(\omega)+g(\omega_1)+g(\omega_3)-g(\omega_2), \quad g_c = g(\omega_1)+g(\omega_2)+g(\omega_3)-g(\omega).$$

The equation (13) is the generalized kinetic equation derived for interacting waves in an unstable medium (due to pumping). In Eq. (13), the usual δ-functions, which ensure the wave vector conservation in the collision integral (right-hand side), are substituted by Lorentzians, in which the gain g is presented. It is a manifestation of the system non-homogeneity in z that is caused by the gain. Other properties of the generalized kinetic equation are close to one of the usual wave kinetic equations. For example, an integral over ω of the collision integral is equal to zero. This is a consequence of the conservation law of wave action (number of waves), which is valid without gain.

Note that similar kinetic equation with Lorentzians was found in the paper [7] where finite wave damping was taken into account. However, there is a principal and fundamental difference. In our case one cannot substitute the Lorentzians by δ-functions (as it was made in the cited work) even for the case of narrow Lorentzians since then the collision integral becomes zero due to complete integrability of the $1D$ Schrodinger equation. This makes our result special and very different.

It is instructive to compare our generalized equation with usual kinetic equation for weak wave turbulence [5]. The latter has two types of solutions: Equilibrium solution and flux solution, both with power spectra. In our case, the collision integral is non-zero, since

it should be balanced by some additional term appearing due to the space inhomogeneity of the system. That leads to a z-dependent characteristic spectrum width. Formally, it is a consequence of the “locality” property of our collision integral (it is determined by frequencies of the order of the external frequency). The “locality” property is also characteristic of the collision integral in usual weak wave turbulence. Our results are not limited to optics, but are relevant to many other areas of science in which the non-linear Schrödinger equation is a master model and four-wave mixing of a large number of waves occurs, such as in ocean waves science [14].

It is possible to substitute $g \to g_0$ in the collision integral since we assumed $aP\Delta^2 \ll g_0$ and explained relative shortness of the non-linear stage (where the collision integral is relevant). However, generally, we should generally keep the term $aP\omega^2$ in the left-hand side of Eq. (13), since it is relevant at the linear stage. As a result, we find

$$\left(\partial_z - 2g_0 + 2aP\omega^2\right) F = \gamma^2 \int \frac{d\omega_1 d\omega_2 d\omega_3}{(2\pi)^2} \delta(\omega + \omega_1 - \omega_2 - \omega_3)$$
$$\frac{2g_0}{4g_0^2 + \Omega^2} \left[F F_2 F_3 + F_1 F_2 F_3 - F F_1 F_2 - F_\omega F_1 F_3\right]. \quad (14)$$

In the paper we examine the case of relatively strong dispersion (wide spectrum), in which $\beta\Delta^2 \gg g_0$ (here, Δ is the spectrum width). The inequality $\beta\Delta^2 \gg g_0$ means that we can pass to the limit of small g in Eq. (13) or (14). However, we should be careful because of the above-mentioned cancellations. In the limit $g_0 \to 0$, the Lorentzian in the collision integral (the right-hand side of Eq.(14)), turns to δ-function of Ω, acquiring the form of the usual collision integral [5]. However, in this limit, the collision integral turns to zero. It is a consequence of the complete integrability of the one-dimensional non-linear Schrödinger equation. An existence of an infinite number of integrals of motion in this case leads to an absence of kinetics in all orders in non-linearity [6].

Therefore, we should go beyond the zero order in g_0 (that gives δ-function) and keep the first order in g_0. Thus we can neglect g_0 in comparison to Ω in the denominator in Eq. (14) and keep g_0 in the nominator to obtain

$$\left(\partial_z - 2g\right) F(z) = \frac{2g_0\gamma^2}{\beta^2} \int \frac{d\omega_1 d\omega_2 d\omega_3}{(2\pi)^2} \delta(\omega + \omega_1 - \omega_2 - \omega_3)$$
$$(\omega^2 + \omega_1^2 - \omega_2^2 - \omega_3^2)^{-2} \left(F F_2 F_3 + F_1 F_2 F_3 - F F_1 F_2 - F F_1 F_3\right). \quad (15)$$

Note the presence of the singular denominator in Eq. (15). This does not lead to any divergency because of integrability (any divergency would mean that the coefficient at the δ-function is not zero). The equation is a starting point of subsequent calculations.

As it follows from Eq. (15), in the linear approximation

$$F_{\rm lin}(z,\omega) \propto \exp\left[2\int dz\; (g_0 - aP\omega^2)\right]. \qquad (16)$$

The expression describes the exponential growth of the signal amplitude. Besides, the relation (16) shows that in the linear regime, the laser spectrum becomes narrower following the gain spectral shape $g(\omega)$. If factor $\mathcal{A} > \Delta_0^{-2}$, in which $\mathcal{A} = \int dz\; aP$ and Δ_0 are the initial spectrum width at $z = 0$. The spectrum width Δ at the end of the linear stage can be estimated as $\Delta \sim \mathcal{A}^{-1/2}$. Note that the spectral width in this case does not depend on the initial spectral width at $z = 0$.

Supplementary Note 3. Diagrammatic expansion

The generalized kinetic equation (13) is derived in the lowest order of the perturbation theory. To examine higher-order corrections, we need to use more powerful tool that is the Wyld diagrammatic technique. Here, we outline this technique, as applied to the considered problem. For illustration purposes, we will re-derive the generalized kinetic equation (13) using the diagrammatic technique.

Correlation functions of the field ψ governed by the equation (1) can be calculated using the diagrammatic technique of the type developed first by Wyld [3] in the context of hydrodynamic turbulence. In the technique, the correlation functions are presented as a series over the non-linearity in the dynamic equation for the field (over γ). We use the version of the formalism developed in works [8, 9, 10, 11] in which the Wyld diagrammatic technique is reduced to a quantum field theory.

The correlation functions are then calculated as functional integrals over the fields ψ, $\psi^\star$ and auxiliary fields p and $p^\star$ with the weight $\exp(-A)$, in which A is the "action"

$$A = \int_0^L dz \int dt \left\{ p^\star \left[(\partial_z - \hat{g})\psi + i\partial_t^2\psi + i\frac{\gamma}{2}\psi|\psi|^2 \right] \right.$$
$$\left. + p\left[(\partial_z - \hat{g})\psi^\star - i\partial_t^2\psi^\star - i\frac{\gamma}{2}\psi^\star|\psi|^2 \right] \right\}. \qquad (17)$$

(Without losing generality, this section uses $\beta = 1$.) Note that an integration over the auxiliary fields guarantees validity of the equation (1) and the complex conjugated equation.

The perturbation expansion is introduced for the pair correlation function

$$\langle \psi(z,t)\psi^\star(z',t')\rangle = \int \frac{d\omega}{2\pi} \exp[-i\omega(t-t')] F(z,z',\omega), \qquad (18)$$

and for the Green functions

$$\langle \psi(z,t) p^\star(z',t')\rangle = G(z,z',t-t'), \tag{19}$$

$$\langle \psi^\star(z,t) p(z',t')\rangle = \bar{G}(z,z',t-t'). \tag{20}$$

Due to space inhomogeneity, the pair correlation function (18) depends on both coordinates (the correlation function introduced in the previous section is taken at coinciding points, $z = z'$). The functions (19,20) determine a linear response of the system to an external influence. Therefore they should possess corresponding causality properties. We formulate dynamics in terms of z, therefore $G(z,z',t) = 0 = \bar{G}(z,z',t)$ if $z < z'$. Note that the correlation functions of the type $\langle pp^\star \rangle$ are zero.

In the framework of the perturbation theory, the functions F and $G, \bar{G}$ are represented as infinite series over γ, in which different terms are determined by bare functions (obtained at $\gamma = 0$). The bare values of the Green functions, G_0 and $\bar{G}_0$, are written as

$$G_0 = \theta(z-z') \int \frac{d\omega}{2\pi} \exp\left[-i\omega(t-t') + \left(g + i\omega^2\right)(z-z')\right], \tag{21}$$

$$\bar{G}_0 = \theta(z-z') \int \frac{d\omega}{2\pi} \exp\left[i\omega(t-t') + \left(g - i\omega^2\right)(z-z')\right]. \tag{22}$$

After substitution $g = g_0 - aP\omega^2$, the integrals (21,22) can be taken explicitly. Note that $G, \bar{G}$ grow exponentially as $z - z'$ increases. The bare value of the function F is determined by the boundary condition (3).

It is convenient to introduce a graphical representation. The Green functions are depicted by double lines directed to complex conjugated fields

$G =$ 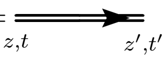

$\bar{G} =$ 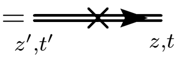

(23)

The bare Green functions G_0, $\bar{G}_0$ are depicted by single lines

$G_0 =$ 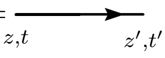

$\bar{G}_0 =$ 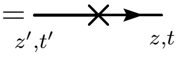

(24)

The pair correlation function (18) is depicted by a wavy line

$F(z,z',\omega) =$ 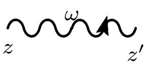

(25)

To construct the perturbation expansion for $D, G, \bar{G}$, we must expand the exponent $\exp(-A)$ in the respective functional integrals in the series over the interaction term

$$A_{\rm int} = \frac{i\gamma}{2} \int_0^L dz \int dt\, \left(p^\star \psi |\psi|^2 - p\psi^\star |\psi|^2\right), \tag{26}$$

and then express the terms of the expansion via the bare objects (which can be done explicitly). The terms of the expansion can be depicted as Feynman diagrams with vertices of the fourth order (corresponding to the factor γ).

A partial summation of the diagrams leads to the following formally exact relations

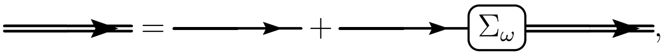

(27)

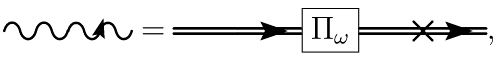

(28)

which is characteristic of the Wyld technique. Here, the blocks Σ (self-energy function) and Π_ω (polarization operator) are subjects of calculation. The quantities in the first non-trivial order are given by two-loop diagrams (that can be obtained in the second order in $A_{\rm int}$)

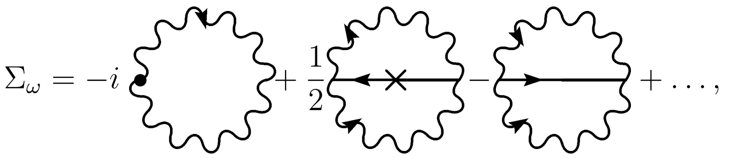

(29)

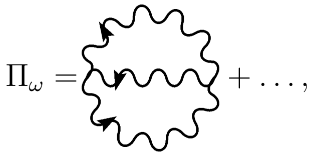

(30)

The equation (27) can be analytically rewritten as

$$[G_0^{-1} - \Sigma(\omega)]G(\omega) = \delta(z - z'), \tag{31}$$

$$G_0^{-1} = \partial_z - g - i\omega^2. \tag{32}$$

A similar relation is valid for $\bar{G}$. Next, applying to the equation (28) the operator G_0^{-1} we find

$$\left(\partial_z - g - i\omega^2\right) F(z, z', \omega) = \int\limits_0^z dy \left[\Sigma(z, y, \omega)F(y, z', \omega) + \bar{G}(y, z', \omega)\Pi(z, y, \omega)\right]. \tag{33}$$

An analogous equation can be obtained for the second argument, z'. It follows from Eq. (33) that if the z-interval is small enough and if the non-linearity is weak then

$$F(y, z, \omega) \approx F(z, z, \omega) \exp\left[\left(i\omega^2 + g\right)(y - z)\right], \quad F(z, y, \omega) = \bar{F}(y, z, \omega). \tag{34}$$

Summing the equation (33), and a similar one for z', we obtain

$$(\partial_z - 2g)\, F(z,z,\omega) = \int_0^z dy\, \left[\Sigma(z,y,\omega)F(y,z,\omega) + G(z,y,\omega)\Pi(y,z,\omega) + c.c.\right], \qquad (35)$$

in which c.c. means a complex conjugated quantity. The next step is substituting into Eq. (35) expressions corresponding to two-loop diagrams (29) and (30) as Σ and Π. We then use the expressions (21,22) for the Green functions and reduce two-point pair correlation functions F to single-point ones by using Eq. (34). We can then calculate the integral over y in Eq. (35) to obtain the equation (13).

Supplementary Note 4. Solution

The right-hand side of Eq. (15) can be estimated as $g_0 F(\gamma I/\beta\Delta^2)^2$. We first analyze the case in which $\gamma I \ll \beta\Delta^2$ at the end of the fibre. That means that the inequality is satisfied everywhere because I monotonically grows as z increases. The inequality $\gamma I \ll \beta\Delta^2$ means that the linear term $2g$ in the left-hand side of Eq. (15) is larger than the collision integral (right-hand side of the equation). The main contribution to the F-evolution by the collision integral is produced at the nearest to the fibre end interval of the length $\sim g_0^{-1}$.

To calculate the non-linear (collision) contribution to $F(L)$, we can exploit the linear law (16) (in which the term with a can be neglected) to obtain $F(z) = \exp[2g_0(z-L)]F(L)$. Then in accordance with Eq. (15), the non-linear correction to F can be written as

$$F_{non} = \frac{\gamma^2}{3\beta^2}\int \frac{d\omega_1 d\omega_2 d\omega_3}{(2\pi)^2}\delta(\omega+\omega_1-\omega_2-\omega_3)$$
$$(\omega^2+\omega_1^2-\omega_2^2-\omega_3^2)^{-2}\left(FF_2F_3 + F_1F_2F_3 - FF_1F_2 - FF_1F_3\right), \qquad (36)$$

in which all functions, $F, F_1, \ldots,$ are taken at $z = L$.

To achieve a statistically steady state, we must satisfy the relation (7). We assume here that the signal scattering is produced by impurities. The reflection coefficient R then weakly depends on the frequency ω, because the impurity size is much smaller than the wavelength. In this case the only parameter coming into the game is $\kappa = |R_\omega|^2 \ll 1$. It then follows from Eq. (7)

$$F(0,\omega) = \kappa F(L,\omega). \qquad (37)$$

To satisfy Eq. (37), we must assume that the $a\omega^2$-contribution to the law (16) is small. Therefore:

$$\kappa \exp\left(2\int_0^L dz\ g_0\right) = 1+\eta, \qquad (38)$$

in which $\eta \ll 1$.

Using the relations (16,36,38), we find from the condition (37)

$$(\eta - 2\mathcal{A}\omega^2)F + \frac{\gamma^2}{3\beta^2}\int \frac{d\omega_1 d\omega_2 d\omega_3}{(2\pi)^2}\delta(\omega+\omega_1-\omega_2-\omega_3)$$

$$\frac{1}{(\omega^2+\omega_1^2-\omega_2^2-\omega_3^2)^2}\left[FF_2F_3+F_1F_2F_3-FF_1F_2-FF_1F_3\right]=0, \tag{39}$$

in which all functions are taken at $z = L$ and $\mathcal{A} = \int_0^L dz\ aP$. As follows from Eq. (39), the spectrum width is determined by the balance of the terms in the left-hand side:

$$\Delta = \sqrt{\frac{\eta}{2\mathcal{A}}}. \tag{40}$$

Note the smallness of Δ in η. Comparing different terms in Eq. (39), we find $I \sim \beta\eta^{3/2}/(\gamma\mathcal{A})$ and

$$I \sim \frac{\beta}{\gamma}\sqrt{\mathcal{A}}\,\Delta^3. \tag{41}$$

Therefore, $\Delta \propto I^{1/3}$ in this regime.

The equation (39) admits a self-similar substitution

$$F(L,\omega) = \sqrt{\frac{3}{2}}\frac{\beta\eta}{\gamma\sqrt{\mathcal{A}}}\phi\left(\frac{\omega}{\Delta}\right), \tag{42}$$

in which Δ is determined by Eq. (40). The equation (39) then leads to the universal form of the equation for the self-similar function

$$(x^2-1)\phi(x) = \int \frac{dx_2 dx_3}{(4\pi)^2}\,\frac{\phi\phi_2\phi_3+\phi_1\phi_2\phi_3-\phi\phi_1\phi_2-\phi\phi_1\phi_3}{(x-x_2)^2(x-x_3)^2}, \tag{43}$$

in which $x_1 = x_2 + x_3 - x$. The numerical solution of the equation gives the normalization factor

$$\int dx\ \phi(x) \approx 23.8.$$

We see from Eq. (41) that the spectral width Δ grows as the intensity I increases. At some level of pumping, γI becomes of the order of $\beta\Delta^2$. At higher pumping levels the lasing regime completely changes. This regime needs a separate consideration. Our preliminary analysis shows that in this regime, the relation $\gamma I \sim \beta\Delta^2$ is satisfied during the non-linear stage of the generation wave propagation (near the fibre span end). These results will be presented elsewhere.

Using equation (43) the generation spectrum shape can be numerically calculated and different ratios between gain and dispersion, $\lambda = 2g/\beta\Delta^2$, see Fig.2 of the main text. Note that in the limiting case of small dispersion, the generation spectrum of random laser approaches the hyperbolic secant shape. Note that in the case of long Raman fibre

laser with a conventional linear cavity, similar hyperbolic secant spectrum shape was observed and introduced in a phenomenological way in the limiting case of the dispersion much bigger than nonlinearity [2]. The fact that the hyperbolic secant shape of the wave spectrum being derived with the help of classical wave kinetics in [2] conventional fiber laser coincides with the spectrum shape derived within the new approach of active cycled wave kinetics for a very different system (random lasers with no cavity) is intriguing and should be further investigated.

Supplementary Note 5. Experimental verification

To experimentally verify the predictions of the developed nonlinear kinetic theory, we designed a random fibre laser (Supplementary Figure 2).

The random fibre laser is built using 850 meters of a phosphosilicate fibre [12]. We choose the phosphosilicate fibre because of specific Raman gain profile. There is a single Raman gain peak with a spectral shape close to be Gaussian (i.e. parabolic in logarithmic scale). The frequency shift between generation wave and pump wave is about 1,330 cm^{-1}. The full width at half the maximum of the gain profile is around 8 nm. Under pumping at 1,115 nm, the laser generates at 1,308 nm. We use a random fibre laser configuration with a broadband mirror of a reflectivity close to 100% from one fibre end and only random feedback from other fibre end. The pump wave is coupled from the free fibre end. We use following fiber parameters (in accordance with notations in eq.(1) and (2)): linear losses $\alpha = 0.09\,\mathrm{km}^{-1}$, dispersion $\beta = 4.3\mathrm{ps}^2\mathrm{km}^{-1}$, Kerr coefficient $\gamma = 7$ $(\mathrm{km}\cdot\mathrm{W})^{-1}$, Raman gain coefficient $g_R = 0.68$ $(\mathrm{km}\cdot\mathrm{W})^{-1}$, coefficient $a = 0.026\,\mathrm{ps}^2\mathrm{W}^{-1}\mathrm{km}^{-1}$.

Due to the symmetry, the configuration with a broadband mirror placed at z=0 is equivalent to the configuration of the laser having twice a fibre length, no point-based reflectors, and pumped from both fibre ends (Supplementary Figure 2b), see review [1] for details. Using a balance equations set [1], we can model the pump and generation power distributions. Generation waves are strongly amplified in the small region near fibre ends. Thus, the main contribution to the backscattered wave also occurs only in the same region near fibre ends. As a result, the theoretical assumption that the feedback in the system is provided by an effective point-based reflector, $I_-(L) = R_{\mathrm{eff}} I_+(L)$, with some random reflection coefficient $R(\omega)$, is well-justified under our experimental conditions. Here, I_+ and I_- are intensities of forward- and backward-propagating waves (see Supplementary Figure 1). The laser is designed in the way that the generation power satisfies the inequal-

ity $(\gamma I_{\text{out}})^2 / \left((g_R P_{\text{in}})^2 + (0.5\beta_2\Delta^2)^2\right) < 1$ in a sufficiently broad range of pump powers above the threshold, allowing use of the perturbation theory for kinetic equation.

Supplementary Note 6. Linear kinetic theory near the generation threshold: Schawlow-Townes limit

The wave kinetic approach developed here for active cyclic systems provides an interesting possibility to expand the seminal result by Schawlow-Townes. It also describes how, after the initial linear spectral narrowing, the wave spectrum of random fibre laser becomes broader due to the nonlinear interactions, when power increases well above the generation threshold. It is common knowledge laser physics that the lasing spectrum becomes *narrower* while the pump power increases inversely proportional to the power [13]. This is true when there are no nonlinear interactions [13]. In our experiments, both spectrum narrowing at low powers and nonlinear spectrum broadening at high power are observed (see Fig. 3b in the main text).

At very low pump power $g_R PL \ll 1$, the emission spectrum shape repeats the spectral shape of the gain profile, which we define in this section as a generalized form of (2)

$$g(\omega) = g_R P e^{-\omega^2/\Gamma_R^2} \tag{44}$$

with a width $\Gamma_R^2 = g_R/a$, in which a is defined in (2). Note that we don't include linear losses α into the definition of $g(\omega)$ (44) for the sake of further simplicity.

Further, we use a configuration from Supplementary Figure 2a with a highly reflective mirror. The starting point of the consideration is the power balance equation on the spectrum of the backward wave I_ω^- propagating from the output $z = L$ towards the mirror $z = L/2$

$$-\frac{dI_\omega^-}{dz} = 2(g - \alpha)I_\omega^- + 4g\hbar\omega_0 + \varepsilon I_\omega^+ \tag{45}$$

with a zero initial condition. Here, ε is a Rayleigh scattering coefficient, $\varepsilon = 0.001 \cdot \alpha$, [1], $4g\hbar\omega_0$ is the term considering energy input from spontaneous emission with Planck constant $\hbar$ and carrier angular frequency is ω_0. We remind that frequency ω is frequency detuning from the center of the gain profile.

The wave propagating in the forward direction (from $L/2$ to L) obeys a similar equation, but we can neglect a Rayleigh backscattering, as it has a small impact on a wave of high enough intensity. Thus, we can describe its evolution as an exponential growth due

to Raman amplification

$$\frac{dI^+_\omega}{dz} = 2(g-\alpha)I^+_\omega + 4g\hbar\omega_0 \qquad (46)$$

Using its approximate solution $I^+_\omega(z) = I^{out}_\omega e^{2(g-\alpha)(z-L)}$ in (45), and relying on boundary conditions at $z = L/2$: $I^+_\omega(L/2) = I^-_\omega(L/2)$, we can finally obtain the formula for the output spectrum I^{out}_ω

$$I^+_\omega(L) = 2\hbar\omega_0 \frac{g(e^{2(g-\alpha)L/2}-1)}{g-\alpha} \frac{e^{2(g-\alpha)L/2}+1}{1-\frac{\varepsilon(e^{4(g-\alpha)L/2}-1)}{4(g-\alpha)}} \qquad (47)$$

The equation (47) defines the spectrum shape and spectral width in Schawlow-Townes limit. We can numerically find the wave spectrum shape (Supplementary Figure 3a), and determine the spectral width from it (Supplementary Figure 3b).

Note that the lasing threshold defined by random distributed feedback is clearly marked on this graph at generation power around 0.01 W by abrupt changes in the spectrum narrowing law. As a result, the spectrum becomes narrower inversely proportional to the generation power, similar to the Schawlow-Townes law [13]. Therefore, we extend Schawlow and Townes' approach to describe spectrum narrowing in random fibre lasers near the generation threshold.

Supplementary References

[1] Turitsyn, S.K. *et al.* Random distributed feedback fibre lasers, *Phys. Reports* in press (2014).

[2] Babin S.A., Churkin D.V., Ismagulov A.E., Kablukov S.I. & Podivilov E.V. FWM-induced turbulent spectral broadening in a long Raman fiber laser. *J. Opt. Soc. Am. B* **24**, 1729-1738 (2007).

[3] Wyld, Jr., H. W. Formulation of the theory of turbulence in an incompressible fluid. *Ann. of Physics* **14**, 143-165 (1961).

[4] Lifshitz E.M. & Pitaevskii, L.P. *Physical kinetics* (Pergamon, Oxford, 1981).

[5] Zakharov, V., L'vov, V. & Falkovich, G. *Kolmogorov Spectra of Turbulence I: Wave Turbulence* (Springer-Verlag, Berlin, 1992).

[6] Zakharov, V.E. Turbulence in Integrable Systems, *Studies in Applied Mathematics* **122**, 219пїЅ234 (2009).

[7] Dyachenko, S., Newell, A.C., Pushkarev, A. & Zakharov, V.E. Optical turbulence: weak turbulence, condensates and collapsing filaments in the nonlinear Schrödinger equation. *Physica D: Nonlinear Phenomena* **57(1)**, 96-160 (1992).

[8] Martin, P. C., Siggia, E. D. & Rose, H. A. Statistical Dynamics of Classical Systems. *Phys. Rev. A* **8**, 423-437 (1973).

[9] de Dominicis, C. Techniques de renormalization de la théorie des champs et dynamique des phénomènes critiques. *J. Phys. Colloques* **37**, C1.247-C1.253 (1976).

[10] Janssen, H. K. Lagrangian for Classical Field and Renormalization Group Calculations of Dynamical Critical Properties, *Z. Phys. B* **23**, 377-380 (1976).

[11] de Dominicis, C. & Peliti, L. Field-theory renormalization and critical dynamics above T_c: Helium, antiferromagnets, and liquid-gas systems. *Phys. Rev. B* **18**, 353-376 (1978).

[12] Dianov, E. M. *et al.* Three-cascaded 1407-nm Raman laser based on phosphorus-doped silica fiber. *Opt. Lett.* **25**, 402-404 (2000).

[13] Schawlow, A. L. & Townes, C. H. Infrared and optical masers. *Phys. Rev.* **112**, 1940-1949 (1958).

[14] Janssen, P. A. E. M. Nonlinear Four-Wave Interactions and Freak Waves, *J. Phys. Oceanogr.*, **33(4)**, 863-884 (2003).
